# Predicting phase behavior of grain boundaries with evolutionary search and machine learning

**DOI:** 10.1038/s41467-018-02937-2

**Published:** 2018-02-01

**Authors:** Qiang Zhu, Amit Samanta, Bingxi Li, Robert E. Rudd, Timofey Frolov

**Affiliations:** 1 Department of Physics and Astronomy, High Pressure Science and Engineering Center, University of Nevada, Las Vegas, NV 89154 USA; 2 Lawrence Livermore National Laboratory, Livermore, CA 94550 USA; 3 Department of Computer Science, University of California Davis, Davis, CA 95616 USA

## Abstract

The study of grain boundary phase transitions is an emerging field until recently dominated by experiments. The major bottleneck in the exploration of this phenomenon with atomistic modeling has been the lack of a robust computational tool that can predict interface structure. Here we develop a computational tool based on evolutionary algorithms that performs efficient grand-canonical grain boundary structure search and we design a clustering analysis that automatically identifies different grain boundary phases. Its application to a model system of symmetric tilt boundaries in Cu uncovers an unexpected rich polymorphism in the grain boundary structures. We find new ground and metastable states by exploring structures with different atomic densities. Our results demonstrate that the grain boundaries within the entire misorientation range have multiple phases and exhibit structural transitions, suggesting that phase behavior of interfaces is likely a general phenomenon.

## Abstract (web summary)

The atomic structure of grain boundary phases remains unknown and is difficult to investigate experimentally. Here, the authors use an evolutionary algorithm to computationally explore interface structures in higher dimensions and predict low-energy configurations, showing interface phases may be ubiquitous.

## Introduction

Properties of structural and functional materials are strongly influenced by the presence of internal interfaces called grain boundaries, which are inherited from materials synthesis and processing. Recent years have seen a rapid growth of evidence suggesting that grain boundaries can exist in multiple states or phases, referred to as complexions^1–4^ and exhibit first-order transitions, marked by discontinuous changes in properties like segregation, mobility, cohesive strength and sliding resistance^4^. These discontinuous transitions were observed in isolated bicrystals with a single well-defined grain boundary as well as in polycrystalline samples with many different grain boundaries. For example, measurements of Ag impurity diffusion in the Σ5(310)[001] grain boundary (GB) in Cu revealed an unusual non-Arrhenius behavior of the diffusion flux characterized by two distinct slopes at low and high temperatures^5^. In polycrystals, studies of doped ceramics demonstrated the non-Arrhenius behavior of growth rate constant which exhibits multiple discontinuous transitions with temperature^2,3^. High-resolution transmission electron microscopy (HRTEM) analysis of these ceramics identified GB structures resembling intergranular films of different thickness^4^. The discontinuous nature of these transitions in polycrystalline materials is somewhat unexpected. If the changes in the grain growth behavior were indeed triggered by transformations of the interface structure, one would expect more gradual changes in properties, since at different interfaces in the material the transitions should take place at different temperatures and impurity concentrations. The discontinuous character of the mobility jumps measured in the experiments on the other hand suggests that the transitions at different interfaces may happen in a more uniform manner.

To explain this puzzling behavior it was proposed that grain boundaries can exist in multiple states called complexions^1–4^. Complexion types are characterized by different amounts of impurity segregation. Monolayer, bilayer, trilayer and thicker films types of complexions have been suggested^3^. Grain boundary complexions were predicted by earlier theoretical work. Phase field models have led to predictions of a variety of first-order and higher order premelting type transitions and mapped them onto bulk phase diagrams^6,7^. More recently, layering transitions associated with GB segregation were investigated using lattice gas models^8–10^ and first-principles calculations^11^. The transitions between complexions of different types could be responsible for changes by orders of magnitude in the grain growth constant with doping. Experimental studies suggested a potential role of complexions transitions on abnormal grain growth in ceramics^3^, activated sintering^12^, and liquid metal embrittlement^13^. More recently the notion of GB complexions has been extended to lattice dislocations, pointing out that they can also exist in multiple states called linear complexions^14,15^.

The body of experimental work currently available on grain boundary phase transitions has raised a number of fundamental questions concerning the atomistic structure of the different phases, the kinetics of the transitions, and the ways in which these interfacial processes influence grain boundary mobilities, diffusivities and mechanical strength. While the experimental investigation of the role of grain boundary phase transitions on materials properties is currently a highly active field of research in the area of structural and functional materials^1,4,13,15–19^, the atomic structure of these grain boundary phases remains unknown. Direct experimental observations of interfacial phase transitions at high temperature by HRTEM are extremely difficult due to inherent limitations^20^. A large number of HRTEM studies of grain boundaries in doped metallic and ceramic materials demonstrated grain boundary structures resembling inter-granular films of different thickness^3,4,13,16,21^. Unfortunately these HRTEM images often do not provide sufficient information about the atomic level structure of these boundaries, so it is still debated whether these grain boundaries are ordered, partially ordered, amorphous or liquid.

On the other hand, atomistic simulations can be used to predict the atomic structure of interfaces and study their thermodynamic and kinetic properties. Despite the decades of extensive modeling research, until recently atomistic simulations did not provide much evidence of first-order grain boundary phase transitions^22^. Recently, the investigation of two high-angle boundaries Σ5(210)[001] and Σ5(310)[001] in Cu demonstrated that the critical impediment to observe such transformations was rooted in inadequate simulation methodology that uses a constant number of atoms and periodic boundary conditions. High-temperature anneals of these boundaries connected to open surfaces allowed the number of atoms in the grain boundary to vary by diffusion, achieving lower free energy states. The simulations revealed multiple new grain boundary phases of these boundaries characterized by different atomic densities and demonstrated fully reversible first-order transitions induced by temperature, changes in chemical compositions and point defects^23–25^. This modeling approach demonstrated phase behavior of two special high-angle boundaries that have been extensively investigated in the past, suggesting that the entire phenomenon could have been overlooked by modeling due an overly restrictive simulation methodology. This work suggested that the greatest obstacle to observing grain boundary phase transitions in simulations is not their absence in the model systems, but the lack of a robust computational tool that can predict complex grain boundary structures.

In recent years, there have been significant advances in predicting the structures from first-principles^26^. Among them, our approach based on the evolutionary algorithm USPEX has proved to be extremely powerful in different systems including bulk crystals^27^, 2D crystals^28^, surfaces^29^, polymers^30^ and clusters^31^, etc. Extending the method to grain boundaries is logically the next step. There have been a few pioneering works reported in the literature^32–34^. For instance, Chua et al. has developed a genetic algorithm to study both stoichiometric and non-stoichiometric grain boundaries of SrTiO_3_^32^. Following this work, two different methods were used^35,36^ to find new low energy structures in the same system. However, these methods were only designed for a system with a fixed number of atoms and supercell size.

In this work we developed a powerful computational tool based on evolutionary algorithms^27^ that predicts structures of interfaces. The search enables an automated exploration of GB structures with variable number of atoms and variable cell sizes. We demonstrate the robustness and the predictive power of our method by performing a structure search for [001] symmetric tilt boundaries in Cu. The evolutionary search augmented with unsupervised machine learning post-processing analysis reveals new ground states and multiple grain boundary phases. We demonstrate GB phase transitions using molecular dynamics (MD) simulations.

## Results

### Grain boundary structure calculations

The choice of the Cu^37^ grain boundaries as a model system is motivated by discontinuous changes in properties in Σ5(310)[001] Cu grain boundary measured experimentally^5^ and the discovery of multiple phases of this boundary by high-temperature MD simulations^23,38^. The study raised new questions concerning whether these transitions are characteristic of only high-angle special boundaries with low Σ or a more general phenomena. It is also not clear how the crystallographic degrees of freedom such as misorientation angle affect the multiplicity of grain boundary phases and their properties. With the newly developed tool, we aim to identify possible multiple grain boundary phases and recover grain boundary energy as a function of misorientation as well as atomic density.

To make a comparison and illustrate the potential importance of this advanced sampling, we first present the results when the grain boundaries are constructed using the common methodology. In this approach often referred to as the *γ*-surface method, the two misoriented perfect half-crystals are joined together, while sampling relative translations of the grains. The prepared configurations composed of two grains are then statically relaxed. During the relaxation the atoms in the boundary fall into the local minima, which concludes the construction. During the search no atoms are added or removed from the grain boundary core.

**Fig. 1:**
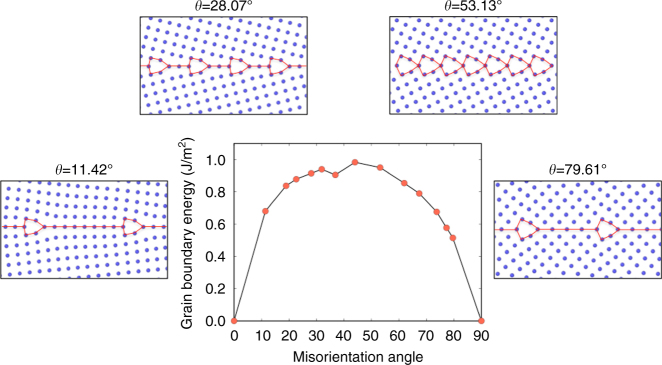
GB structures predicted by the *γ*-surface method. The kite family of grain boundary structures predicted by the conventional simulation methodology. Low-energy grain boundary structures of symmetric tilt boundaries in fcc Cu are composed of Kite-shaped structural units. The units separation distance changes with the misorientation angle *θ*. The construction does not add or remove atoms from the grain boundary core, so not all possible states are sampled. Grain boundary energy of the 13 grain boundaries studied is plotted as a function of misorientation angle

Figure 1 illustrates the well-known lowest energy configurations obtained by this approach^39,40^. The structures of the boundaries are composed of kite-shaped units. The distance between these structural units depends on the misorientations angle *θ*. In the paper we will refer to this family of grain boundary structures as the Kites family. For low-angle boundaries composed of a periodic array of well-separated edge dislocations, the kite-shaped units represent the dislocation core structure. Figure 1 illustrates grain boundary energy as a function of misorientation angle *θ* obtained from the *γ*-surface construction. This conventional methodology generates a large number of distinct grain boundary states with different energies that correspond to different grain translation vectors. However, all are built out of the same fixed number of atoms compatible with the number of atoms in one plane in each of the adjacent crystals. Due to this constraint many potentially lower energy structures that have different atomic density are not sampled. A growing number of recent studies demonstrated that that the *γ*-surface method is often not sufficient to predict true ground states^32,33,41–44^.

On the other hand, the evolutionary search implemented in this work samples very different grain boundary configurations by rearranging atoms within a grain boundary core prior to relaxation, adding and removing atoms from the boundary and changing the dimensions of the grain boundary area on the fly. It is well-known that the complexity exponentially increases with the growing dimensionality^45,46^. In that case, a key to ensure efficient sampling is to find a balance between individual quality and population diversity. Any pure random structure initialization or variation operation is very likely to lead to disordered like structures with close energetics. To address this challenge, we followed the idea of coarse-grained modeling and define the simplified representations during the stage of structure generation. Some key representations used here are symmetry, vibrational modes and degree of local order^47^. The tool generates a population of grain boundary structures and improves them over several generations to predict low-energy configurations. During the evolution complex and diverse structures with different atomic densities are sampled by operations of heredity and mutation (see Supplementary Note 1 and Supplementary Figs. 4 and 5 for details). In a typical search several thousand configurations are generated and their energy is evaluated using empirical force fields. The low-energy configurations are automatically stored and used later for the post-analysis.

**Fig. 2:**
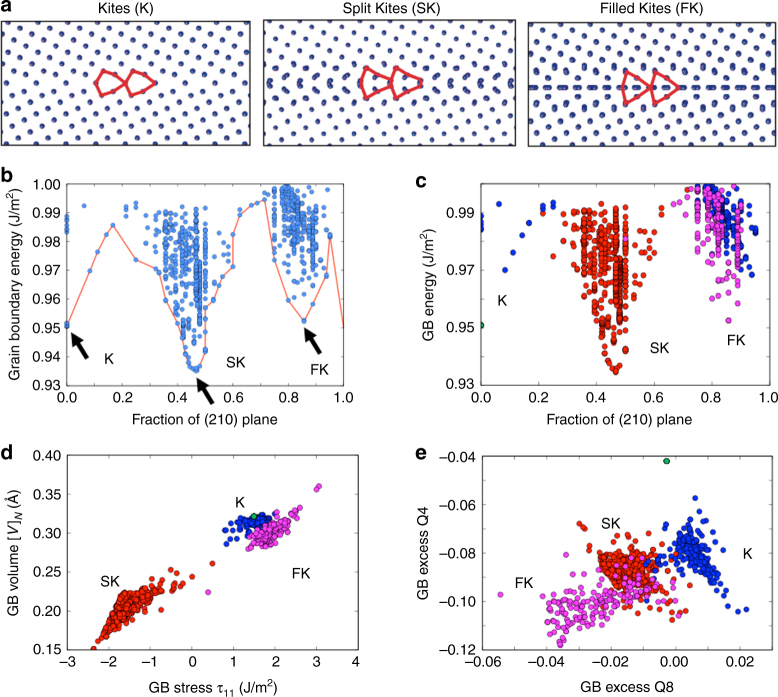
Evolutionary search and clustering analysis identify GB phases. The evolutionary search and clustering analysis identify three grain boundary phases of Σ5(210)[001]. The evolutionary algorithm explores different atomic densities and identifies multiple grain boundary phases: **a** Kites, Split Kites, and Filled Kites. The three phases correspond to the energy minima as a function of number of atoms. **b** Energy of grain boundary configurations generated by the evolutionary search as a function of number of atoms. **d**, **e** The generated structures are automatically clustered into three grain boundary phases according to similarities in their excess properties. **c** Grain boundary energy plot same as in **b** with data points colored according to the clustering

A typical result of the evolutionary search for a Σ5(210)[001] grain boundary is illustrated in Fig. 2b. Because atoms are added and removed from the grain boundary core during the search, the grain boundary energy of different configurations is plotted as a function of the number of atoms in the system, which is measured as a fraction of the number of atoms in a (210) plane. Each point on the plot represents one particular structure generated by the algorithm. The red line connecting the lowest energy configurations for different atomic fractions shows that the grain boundary energy has three distinct minima corresponding to different GB phases called Kites, Split Kites and Filled Kites shown in Fig. 2a. Prior modeling work demonstrated fully reversible transitions between these different grain boundary phases^23–25^. The well-known Kite phase of this grain boundary is composed of the structural units discussed earlier. The structures of the other two phases on the other hand are more complex and are composed of multiple distinct structural units. This structural diversity apparently gives rise to a rich variety of low-energy Split Kite and Filled Kite configurations that have different grain boundary dimensions. For example, nearly degenerate in energy, but distinct Split Kite type structures were found for cross-section sizes ranging from 1 to 25 times of the area of the regular Kites (See Supplementary Fig. 1). This configurational diversity should contribute to the entropy of these grain boundary phases^48–50^ and may have consequences for their high-temperature stability.

### Clustering analysis

The three energy minima shown in Fig. 2a represent the lowest energy configurations of the three grain boundary phases. Other structures generated by the evolutionary search may correspond to variations of these three phases or belong to other grain boundary phases that have not been identified yet. For example, a Kite configuration with a single vacancy or an interstitial will have a different atomic density and energy from that of the perfect Kite structure. However, this defective grain boundary should still be identified with the Kite phase. In general, each grain boundary structure generated by the evolutionary search represents just one microstate. A grain boundary phase on the other hand is a macrostate: it is represented by an ensemble of similar microstates. To identify distinct macrostates, i.e., predict the number of grain boundary phases, we cluster the generated grain boundary structures based on the similarity in their properties. In a single component system a grain boundary is described by a set of excess properties such as excess volume per unit area [*V*]_*N*_, grain boundary stress tensor $\hat \tau ^N$ and the number of atoms [*n*] (See Supplementary Note 2 and Supplementary Fig. 6 for the definitions). First-order phase transitions manifest themselves by discontinuous changes in thermodynamic properties, which in turn suggests that these properties could be used to distinguish different macrostates. In addition to these thermodynamic properties which explicitly enter the equation of state or the adsorption equation^51–54^, we can formally introduce other excesses based on structural order parameters. In this work we use Steinhardt order parameters *Q*_4_, *Q*_6_, *Q*_8_ and *Q*_12_ designed to distinguish different bulk phases based on local environments^55,56^. In our work Q-series were calculated for each atom in the system and the excess grain boundary amounts of [*Q*_*i*_]_*N*_ per unit area were computed as described in the Supplementary Note 2. This new application of the Q-series was developed to capture differences in local environment present in different GB phases. We assign a vector *f* = ([*n*], [*V*]_*N*_, $\tau _N^{11},\tau _N^{22}$, [*Q*_4_]_*N*_, [*Q*_6_]_*N*_, [*Q*_8_]_*N*_, [*Q*_12_]_*N*_) composed of four thermodynamic and four structural features to each grain boundary configuration. A distance between two grain boundary structures *a* and *b* is then calculated as

1 $$d\left( {f^a,f^b} \right) = \mathop {\sum}\limits_{i = 1}^8 \left( {\left( {f_i^a - f_i^b} \right){\mathrm{/}}\left( {f_i^a - f_i^b} \right)_{{\mathrm{max}}}} \right)^2$$

where all the feature differences were renormalized, so that their values are in the range from 0 to 1. With the distance defined, the clustering was performed using the method of fast search and find of density peaks^57^. In this method for each data point we calculate the number of neighbors *ρ*_*i*_ within a cutoff distance *d*_c_ and the minimum distance *δ*_*i*_ from the point to the other point that has a higher number of neighbors. The centers of the clusters are then identified as points that have high number of neighbors and separated from each other by the largest distances. All other data points are then assigned to the closest cluster centers which completes the clustering procedure.

### Clustering results for the Σ5(210)[001] GB

Figure 2 illustrates an example of the clustering analysis performed for the Σ5(210)[001] boundary, which predicts three different grain boundary phases. To visualize the data in the eight-dimensional space of the features we show the data points projected on a plane formed by two different excess properties. Figure 2c reveals a strong clustering of the data points based on properties such as excess volume [*V*]_*N*_ and excess stress *τ*^*N*^. The structures in the red cluster were identified with Split-Kite phase, while the blue and magenta represented Kites and Filled Kites, respectively. Note that the Split-Kite structures have properties very different from both Kites and Filled-Kites. On the other hand, Kites and Filled-Kites phases have relatively similar thermodynamic properties and the excess properties based on order parameters proved useful to distinguish the two phases as shown in Fig. 2d. Overall, Fig. 2 demonstrates that clustering based on multiple GB excess properties can be used to identify distinct grain boundary phases. The analysis also reveals the degree to which the thermodynamic properties can vary within each macrostate, which provide insights regarding the stability of the different grain boundary phases.

### GB energy as a function of *θ* and atomic density

**Fig. 3:**
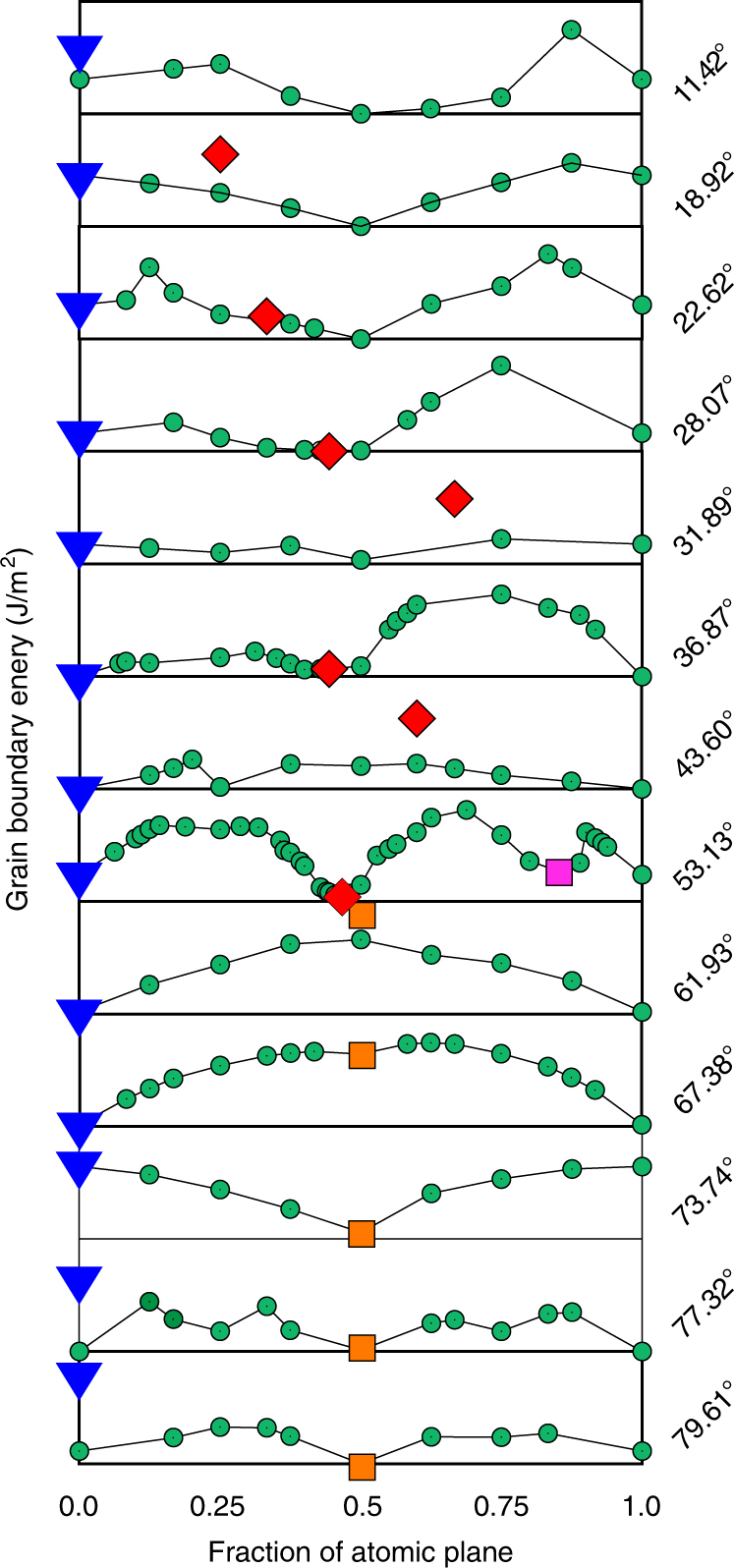
Energy map of GB phases. Evolutionary search and clustering identify new ground states and multiple grain boundary phases. The search explores different atomic densities and finds low-energy grain boundary configurations (green circles) ignored by the conventional methodology. For each grain boundary (angle *θ*) atomic fractions and energies of different grain boundary phases at 0 K are indicated by blue triangles (Kite family), red diamonds (Split Kite family) and orange squares (Extended Kite family)

In contrast to the *γ*-surface construction which assumes that grain boundary energy is a function of misorientation angle *θ* alone, the evolutionary search and the clustering analysis of the Σ5(210)[001] boundary demonstrates the importance of exploring different atomic densities. In this work we reconstruct GB energy as a function of the misorientation angle and number of atoms in the boundary core. Figure 3 illustrates the results of the grand-canonical search spanning the entire misorientation range of symmetric tilt boundaries from 0° to 90°. For each of the 13 grain boundaries studied, the green curves on the plot show the lowest GB energy calculated versus the atomic fraction of the corresponding grain boundary plane. The blue triangles at the origin of the plot correspond to the Kite structures obtained by the *γ*-surface approach that does not add or remove atoms. The plot reveals that within the entire misorientation range the evolutionary search finds new ground states that require a change in the atomic density. Most boundaries within two angle intervals 0° < *θ* < 53.13 and 73.74° < *θ* < 90.0° exhibit at least one strong minimum which is close to about half of the atomic plane fraction. These two intervals are separated by a narrow range of angles around 65° where the grain boundary structures with unconventional density become unfavorable at 0 K. This interval separates grain boundary groups with different structural units. Many boundaries especially in the high-angle range exhibit multiple minima suggestive of multiple grain boundary phases. There are yet other boundaries with misorientation angles of 31.89° and 43.60° that show almost negligible variation in energy with changing atomic density. This behavior suggests that these boundaries can absorb point defects with no energetic penalty and may not be very stable against fluctuation of atomic density.

Low-angle boundaries near the 0° and 90° are composed of periodic arrays of isolated edge dislocations. The evolutionary search results shown in Fig. 3 indicate that the dislocation core structure can be represented by multiple atomic configurations that generally also require grand-canonical optimization: atoms have to be added or removed from the dislocation core. The multiple dislocation core configurations are examples of 1D phases, referred in the recent literature as 1D complexions^15,58^. Different core structures and transitions may have a strong effect on dislocation mobility^14^.

**Fig. 4:**
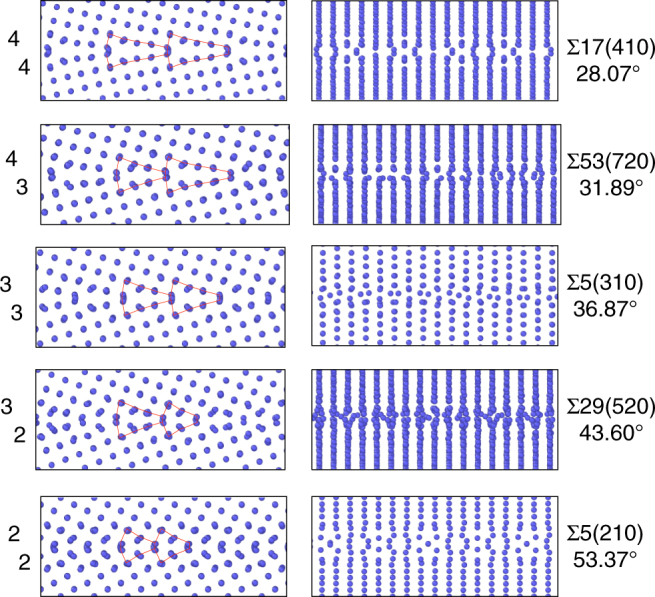
Split Kite family. Split Kite phases of five representative boundaries predicted by the evolutionary search and clustering analysis. For each misorientation GB structures are viewed parallel to the [001] tilt axis (left column) and normal to it (right column). The size of the structural units of this family changes with misorientation. Split Kites have higher atomic density compare to Kites, with extra atoms occupying interstitial positions between [001] planes

Despite the large number of new grain boundary configurations found, this richness of structures is easy to comprehend because they can be grouped into families of structures with similar characteristic units. The Kite family illustrated in Fig. 1 was already introduced with the *γ*-surface approach and has different grain boundaries with similar kite-shaped structural units and atomic density. Our grand canonical evolutionary search identifies two new families of grain boundary phases which we call Split Kites and Extended Kites. In the energy vs. atomic density map in Fig. 3 the three families are indicated by blue triangles (Kites), red diamonds (Split Kites) and orange squares (Extended Kites). Figure 4 illustrates split kite structures for several representative boundaries, which are composed of similar structural units. The structure of these boundaries changes as the misorientation angle increases from *θ* = 28.07° to *θ* = 53.37°. The Σ17(410)[001] at *θ* = 28.07° is composed of units with the size equal to four 1/2[100] lattice spacings. The Σ53(720)[001] which has a higher misorientation angle of 31.89° consists of alternating units with sizes 3 and 4. Other grain boundaries are composed of units with size 3 only, alternating 3 and 2, until at *θ* = 53.37° the Σ5(210)[001] boundary is composed of units with size 2. The Σ5(210)[001] also exists in a Filled Kite structure, which was not found in other 13 boundaries and is likely to be stable in a narrow misorientation angle range around 53.37°.

All Split Kite structures are characterized by higher atomic density relative to Kite family. In the Kite family all the atoms at the boundary are confined to the [100] planes. On the other hand, in all Split-Kite structures additional atoms densely occupy positions in-between the [001] planes, creating complex structures composed of multiple distinct subunits. The atomic arrangement with the boundaries along the tilt axis is illustrated in the right-hand side of Fig. 4. This internal structure gives rise to a rich configurational diversity and may contribute to the entropy of these structures at finite temperature. Notice that in Fig. 3 Split-Kite configurations were not identified with the ground states for some misorientations, see Supplementary Figs. 2 and 3 for further discussion on symmetries of these structures.

**Fig. 5:**
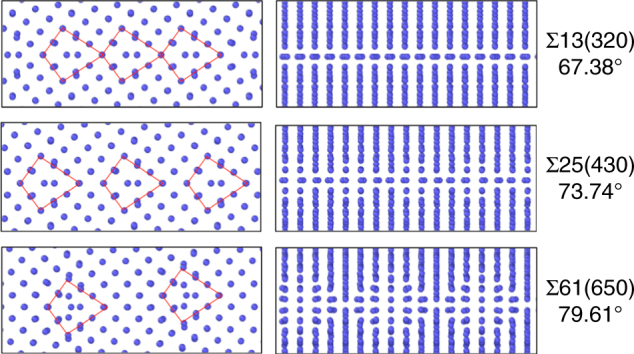
Extended Kite family. Extended Kite phases of three representative boundaries predicted by the evolutionary search at 0 K. The misorientation angles are indicated on the figure. For each misorientation GB structures as viewed parallel to the [001] tilt axis (left column) and normal to it (right column). Extended Kites have higher atomic density compare to Kites, which correspond to half of the atomic plane. The structural units are outlined and change their separation with the increasing misorientation angle

Different structural units appear at misorientation angles *θ* > 53.37° and are illustrated in Fig. 5. The units of the boundary are [110] edge dislocations with more extended dislocation core structure than regular Kites. For this reason we refer to this family of grain boundaries as Extended Kites. Similar to Split Kites, the Extended kites are denser than Kites and become more energetically favorable as the misorientation angle *θ* increases away from 61.93°. The misorientation interval 53.37° < *θ* < 61.93° represents a transition region where both structural units may be equally favorable at some temperature. Grain boundary structure in this misorientation range is likely to exhibit checkerboard pattern composed of both split-kites and extended kites structural units

### GB structures and transitions at finite temperature

**Fig. 6:**
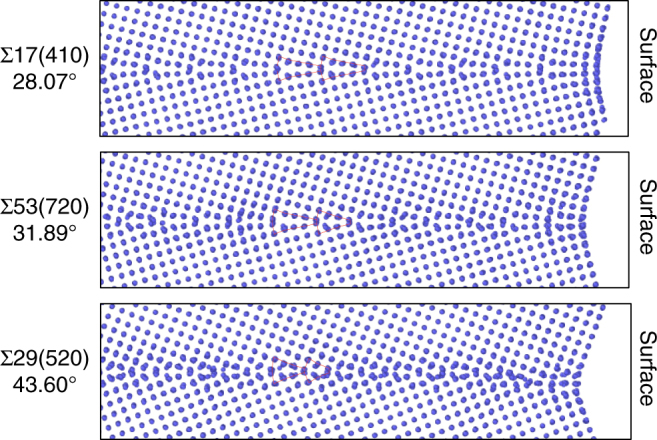
Equilibrium structures at high temperature. High-temperature Split-Kite grain boundary phases of three representative grain boundaries independently predicted by molecular dynamic simulations. Grain boundary phase transitions occur in the simulations, because open surfaces and grain boundary diffusion at 900 K enable variation of the atomic density in the GB core. The structures match the predictions of the evolutionary search at 0 K

To validate the structures predicted at 0 K by the evolutionary search and demonstrate possible grain boundary phase transitions, we performed high-temperature MD simulations of a subset of relatively high-angle boundaries. In these simulations the grain boundaries were terminated at open surfaces following the methodology proposed in ref.^23^. Open surfaces act as sources and sinks of atoms and effectively introduce grand-canonical environment in the grain boundary core. This approach is less effective for low-angle boundaries due to much lower diffusivity normal to the tilt axis. We chose regular kite structures illustrated in Fig. 1 as the initial configurations prior to annealing. During the 900 K anneal for tens of nanoseconds the grain boundaries transformed to Split Kite configurations. Figure 6 illustrates three representative high-angle grain boundaries following the transformation. The high-temperature structure of these boundaries matches the Split Kite configurations independently generated by the evolutionary search. These MD simulations show that the Split Kite family represents the structure of grain boundaries at high temperature and confirm that our structure sampling at 0 K can generate grain boundary phases relevant to finite temperature.

For a number of misorientations we find that that Split Kite structures observed in high temperature MD simulations are not the ground state at 0 K, as illustrated in Fig. 3. These grain boundaries have different structures at low and high temperature and exhibit first-order transitions that result in discontinuous changes in properties, analogous to those reported in the recent experimental studies^5^. For example, Σ5(210)[001] and Σ5(310)[001] exhibit such transitions^23^ and the different GB phases are easy to identify even at 0 K because they correspond to distinct GB energy minima as a function of the number of atoms. On the other hand, in some boundaries such as Σ29(520)[001] and Σ53(720)[001], Split Kite structures do not correspond to such minima and cannot be found within the lowest energy configurations at 0 K. In this case, the clustering analysis becomes invaluable for the identification of the potential high-temperature grain boundary phases.

### Clustering results for the Σ29(520)[001] GB

**Fig. 7:**
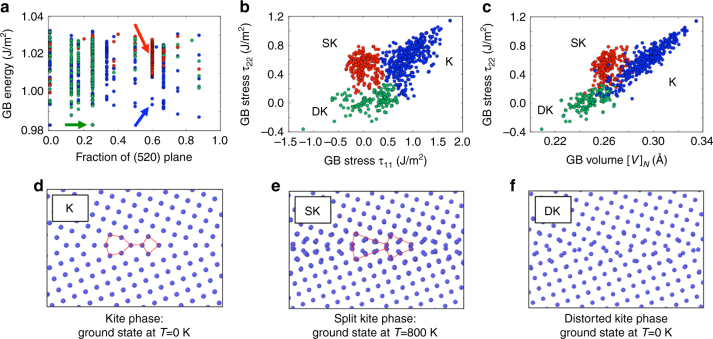
Clustering identifies GB phases observed at high temperature. Metastable grain boundary phases identified by the clustering analysis at 0 K become stable at high temperature. Evolutionary search **a** and clustering **b**, **c** for the Σ29(520) [001] predict three grain boundary phases: **d** Kites, **e** Split Kites, and **f** Distorted Kites. Kite phase is the ground state at 0 K. Split Kite phase is a high energy state at 0 K, but becomes a ground state with the lowest free energy at finite temperature, as demonstrated by a transformation in MD simulations at 900 K

Figure 7a illustrates results of the energy search generated for the Σ29(520)[001] GB by the evolutionary algorithm. Notice that the energy as a function of atomic density shows no obvious minima, like the minima observed for Σ5(210)[001], so it is not clear from this plot alone that this boundary may have multiple phases. Figure panels 6b and c shows the excess properties of the generated structures and the clustering analysis identifies three distinct phases. The representative grain boundary structures from the three different clusters are illustrated in Fig. 7d–f. The red cluster of points corresponds to Split Kite configuration shown in Fig. 7e and observed at high temperature. The majority of the configurations has the atomic fraction of 0.6. The energy plot in Fig. 7a clearly demonstrates that Split Kites represent higher energy state compare to all other configurations even within the subset with the atomic fraction of 0.6. This clustering analysis demonstrates that the examination of the lowest energy configurations alone is not sufficient and will fail to predict the high-temperature GB phases. The clustering analysis captures the heterogeneity in properties of the generated structures and identifies multiple macrostates. Some macrostates may not be the lowest energy configurations at 0 K, but can potentially become the lowest free energy state at a finite temperature or due to varying chemical composition. The evolutionary search and clustering analysis complemented by energy calculations can generate grain boundary phase diagrams and predict grain boundary phase transitions.

## Discussion

Using the advanced evolutionary search and clustering analysis we have uncovered rich phenomena unexplored by previous computational studies of grain boundaries. Based on the successes of applying the evolutionary algorithm in the prediction of bulk crystals, surfaces and clusters, we developed a computational tool to explore the low-energy GB structures in a vast compositional, dimensional, and structural space. The developed algorithm generates a diverse population of configurations while inserting and removing atoms from the grain boundary core and changing grain boundary dimensions. In this work the evolutionary search was applied to reconstruct grain boundary energy surface as a function of both misorientation and atomic density in a model system of Cu symmetric tilt boundaries and predicted new ground states of grain boundaries within the entire misorientation range. For most misorientations multiple grain boundary phases were found demonstrating that phase behavior of interfaces is a general and common phenomena, not limited to few special high-angle boundaries.

The computational discovery of these phases and modeling of the transitions became possible only with the new methodology. Specifically, we designed a clustering procedure that analyses the results of the evolutionary search and automatically identifies different macrostates or grain boundary phases by grouping the individual configurations according to their thermodynamic and symmetry properties. While many studies of structure prediction at 0 K often focus on finding configurations with the lowest energy possible, the clustering analysis examines grain boundary structures within a finite energy interval and identifies multiple metastable grain boundary phases in addition to the ground state. While for some misorientations these metastable states were also the energy minima as a function of atomic density, in general they are just higher energy macrostates that are not minima of energy as a function any particular property and as such were identified only with help of the clustering analysis.

High-temperature MD simulations with open surfaces demonstrated first-order grain boundary transitions between the different grain boundary phases independently predicted by 0 K calculations. This confirms that the ground states and metastable states generated by the evolutionary search and the clustering analysis at 0 K are relevant to the prediction of grain boundary structures at finite temperature. Moreover, in principle the temperature induced grain boundary phase transitions can be predicted by calculating the free energy of the different metastable states using available computational methods^59–62^. Thus in the future, the 0 K search developed in this work augmented with an efficient free energy calculation scheme can be used to construct grain boundary phase diagrams.

In this work we demonstrate that within the entire misorientation range certain types of structures with similar characteristics can be grouped into families of Kites, Split-Kites and Extended-Kites. For example, the characteristic features of the Split-Kite phase is their higher atomic density compared to that of Kites and configurationally more diverse atomic arrangement of the structure. Split-Kites were found to be the high-temperature phases for the majority of grain boundaries studied. The presence of distinct families of grain boundaries like Kites, Split-Kites and Extended-Kites with properties that are different across the entire misorientation range may help explain the sharp discontinuous transitions in mobility observed in polycrystalline materials. For example, an addition of impurities with large size mismatch would stabilize Kite family of grain boundary structures over the much denser Split-Kite and Extended-Kites families in the entire polycrystalline sample. The ability to predict families of phases and their characteristic excess properties may provide guidance on how interfaces with certain structure and properties can be enforced in a material by alloying elements or temperature, ultimately providing a way to achieve the desired materials microstructure and properties.

The insights gained in this work about grain boundaries are also relevant to other lattice defects such as dislocations and triple junctions. Low-angle boundaries near 0 and 90 misorientations studied in this work are composed of rows of edge dislocations. The evolutionary search predicted new ground states of dislocation core structures. The optimization required sampling of different atomic arrangements as well as addition and removal of atoms from the dislocation core. This type of sampling was not typically performed in studies that attempted to predict dislocation structures. It is well known that the core structure can have a pronounced effect on dislocation mobility. The systematic investigation of different dislocation core structures and their properties is subject to future work.

## Methods

### GB structure calculations at 0 K

For each grain boundary we ran 3–5 independent evolutionary searches. Each search evolves over up to 50 generations. The search explores different atomic densities ranging from 0 to 1 measured as a fraction of the number of atoms found in one bulk atomic plane parallel to the grain boundary. We conducted structure searches sampling the entire range of densities as well as searches constrained around certain atomic densities and found that both types of searches are useful. A typical run explores the structures ranging from 500 to 5000 atoms for the entire model and 30–300 atoms for the GB region. For each grain boundary we explore different grain boundary areas by replicating the smallest possible cross-section up to 25 times. See Supplementary Note 1 for more details. The energy of the generated configurations was evaluated with LAMMPS code^63^.

### Finite temperature simulations

Molecular Dynamics simulations were performed in the NVT ensemble with Nose-Hoover thermostat using the LAMMPS code^63^. Periodic boundary conditions were applied only along the [001] tilt axis. In the direction normal the grain boundary plane the simulation block was terminated by two boundary regions in which the atomic positions were kept fixed during the simulation. In the *x* direction the boundaries were terminated by two open surfaces. The dimensions of the simulation block were 50 Å along the tilt axis and 200 Å in the direction normal to the grain boundary plane. In the *x* direction the block size varied from 250 to 350 Å depending on the misorientation angle. Isothermal simulations at *T* = 900 K and *T* = 800 K were performed for 200 ns each.

### Data availability

The data that support the findings of this study are available from the authors upon request.

## Electronic supplementary material

Supplementary Information
