# Supplementary material for: Predicting phase behavior of grain boundaries with evolutionary search and machine learning
Source: Nat Commun. 2018 Feb 1;9:467. doi: 10.1038/s41467-018-02937-2 (PMC5794988; doi:10.1038/s41467-018-02937-2)
Supplement: Supplementary file 1 — Supplementary Information [file 41467_2018_2937_MOESM1_ESM.pdf]

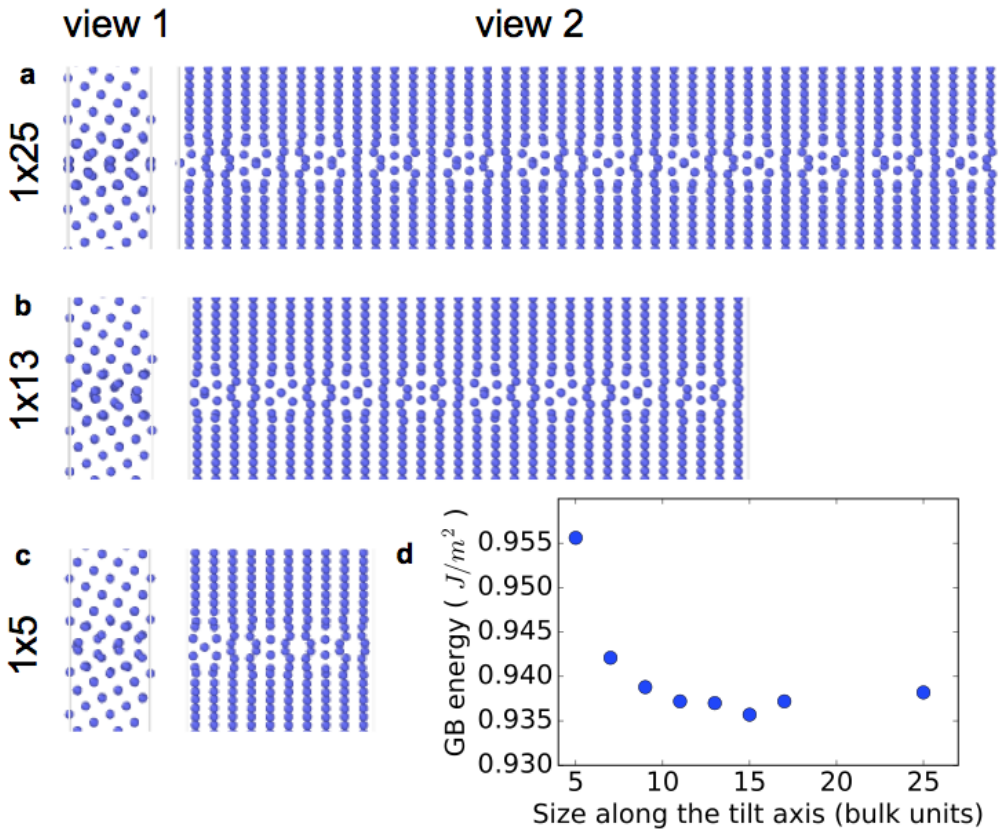

Supplementary Figure 1.

1×25, 1×13 and 1×5 reconstructions of the Split Kite phase of the $\Sigma 5(210)[001]$ grain boundary. In the left-hand side images (view 1) the [001] tilt axis is normal to the plane of the screen. In the right-hand side images (view 2) the [001] tilt axis is parallel to the plane of the screen. The different reconstructions are composed of similar structural units and are nearly indistinguishable to a eye in view 1. Grain boundary energy of different reconstructions as a function of the dimension along the [001] tilt axis. The energy plot demonstrates the need to explore grain boundary areas much larger than the periodic unit of the bulk cell to find the low-energy configurations. Distinct grain boundary configurations with sizes 9 and larger have nearly degenerate energy.

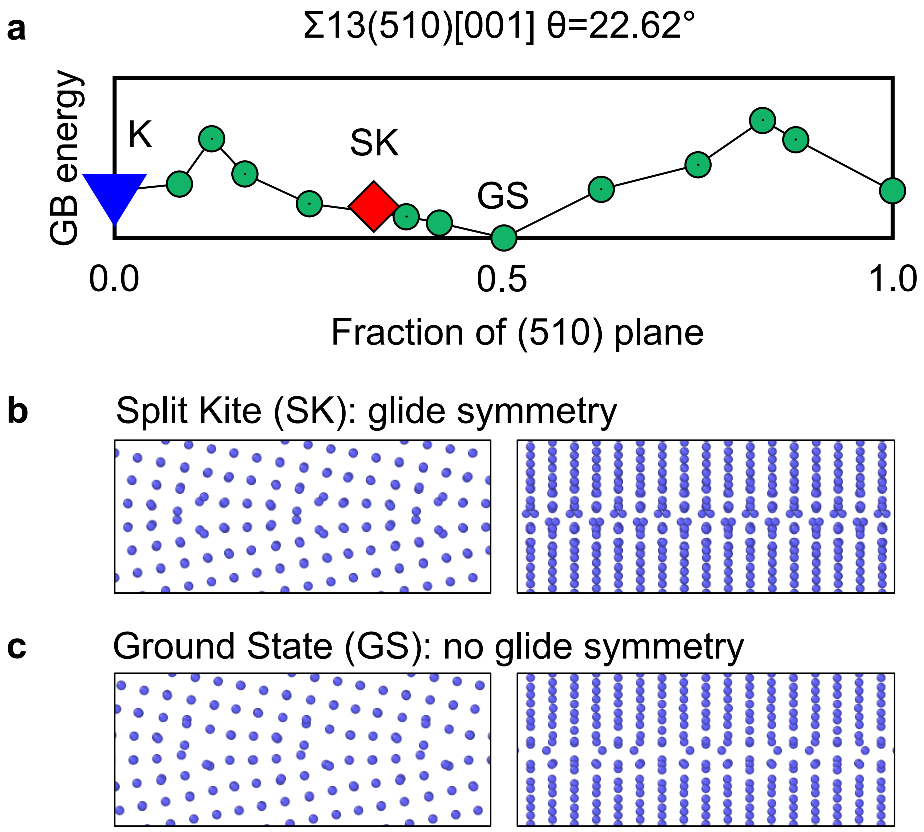

Supplementary Figure 2.

Symmetries of the ground state (GS) and Split Kite (SK) structure of the $\Sigma 13(510)[001]$ grain boundary. (a) Grain boundary energy as a function of atomic fraction measured of (510) plane. (b) We call SK phase grain boundary structure that has glide symmetry, which is not the ground state of $\Sigma 13(510)[001]$ boundary at 0K. (c) Ground state at 0.5 atomic fraction does not have glide symmetry. The panels in (b) and (c) show two different views of the grain boundary structure. Layer group symmetry elements exist in many of the generated grain boundary configurations.

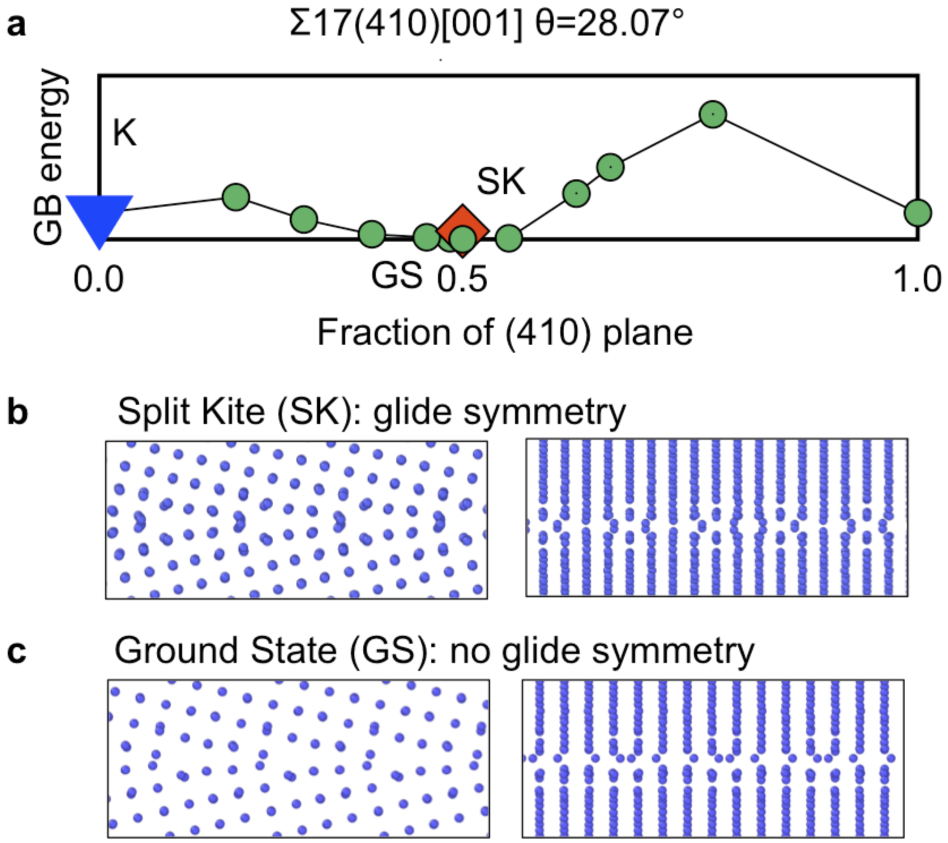

Supplementary Figure 3.

Symmetries of the ground state (GS) and Split Kite (SK) structure of the $\Sigma 17(410)[001]$ grain boundary. (a) Grain boundary energy as a function of atomic fraction measured of (410) plane. (b) We call SK phase grain boundary structure that has glide symmetry, which is not the ground state of $\Sigma 17(410)[001]$ boundary at 0K. (c) Ground state at 0.5 atomic fraction does not have glide symmetry. The panels in (b) and (c) show two different views of the grain boundary structure.

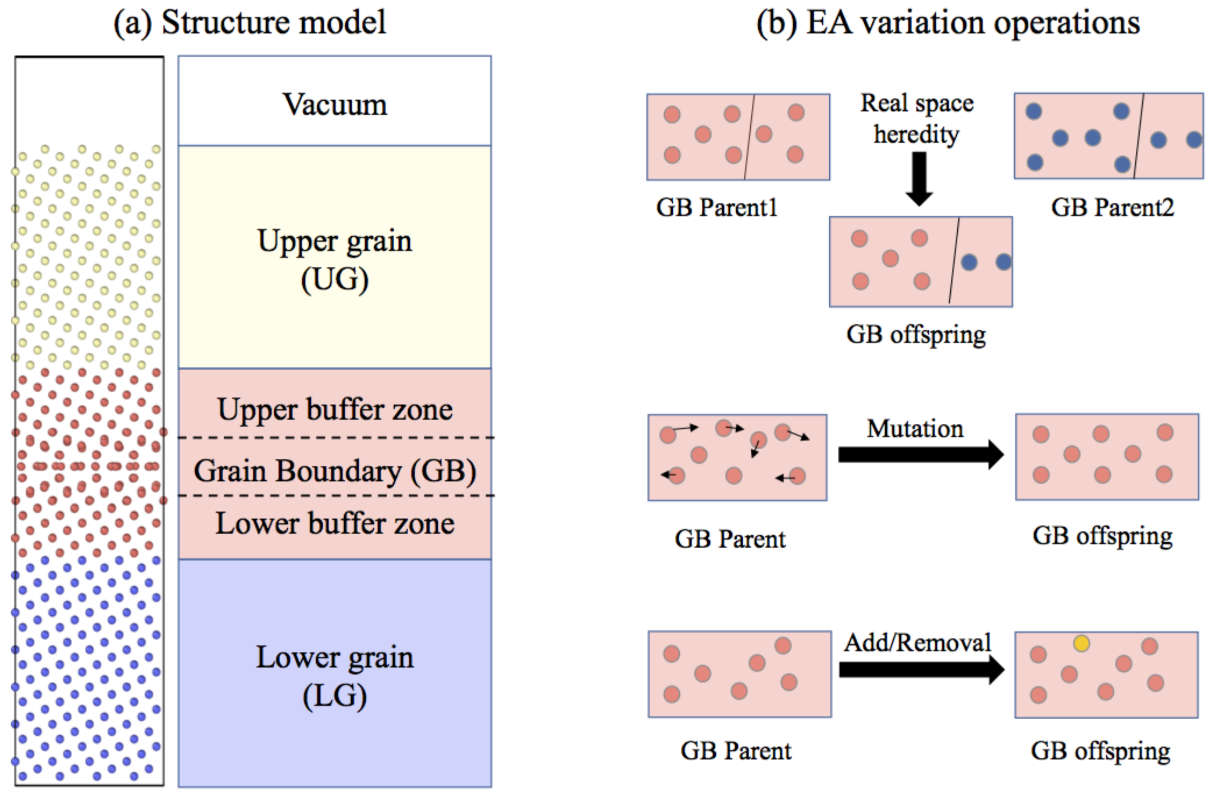

Supplementary Figure 4.

Schematic of the evolutionary algorithm (EA) for grain boundary prediction. (a) the model of GB representation; (b) the scheme of variation operators to generate new offspring in the context of EA.

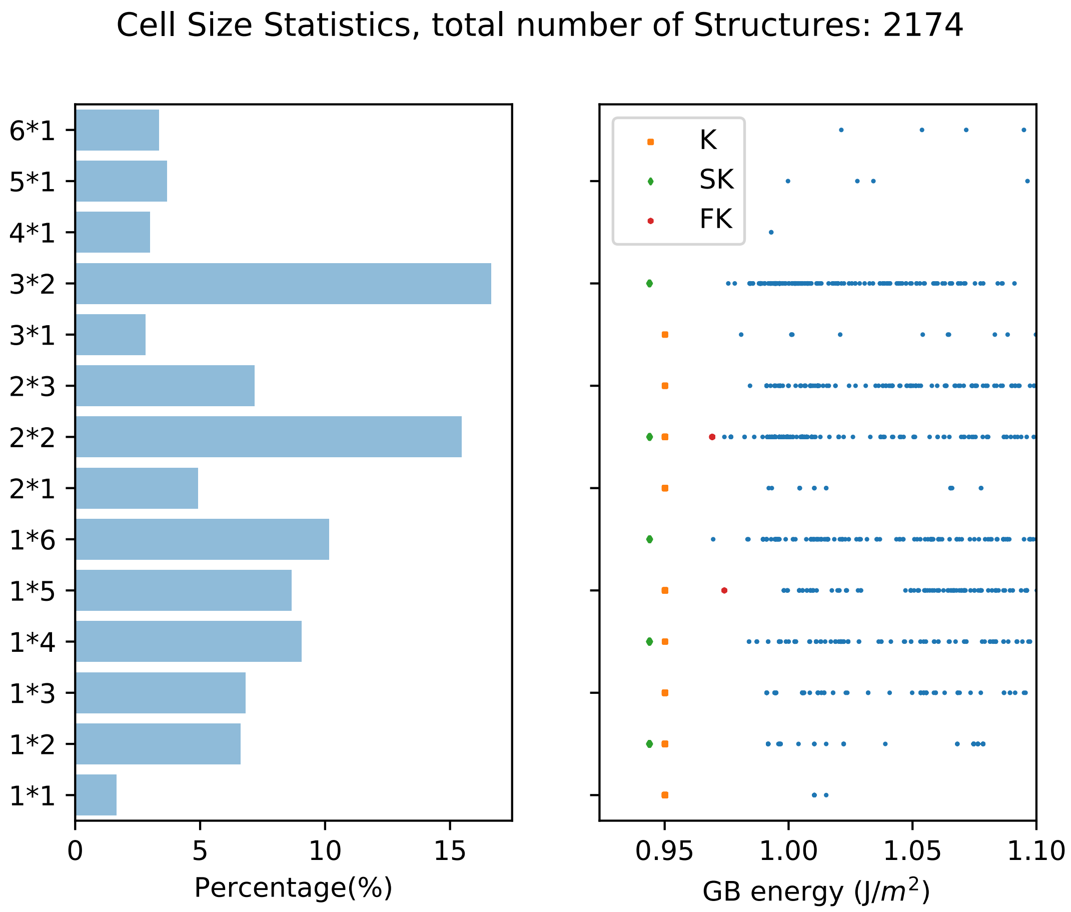

Supplementary Figure 5.

The GB cross-section statistics for a typical structure search for the $\Sigma5(210)[001]$ GB in Cu. (a) The distribution of the sampled cell sizes for the entire search. (b) The energy distribution for all structures categorized by the cell sizes. Each point on the plot corresponds to a structure generated by the evolutionary search.

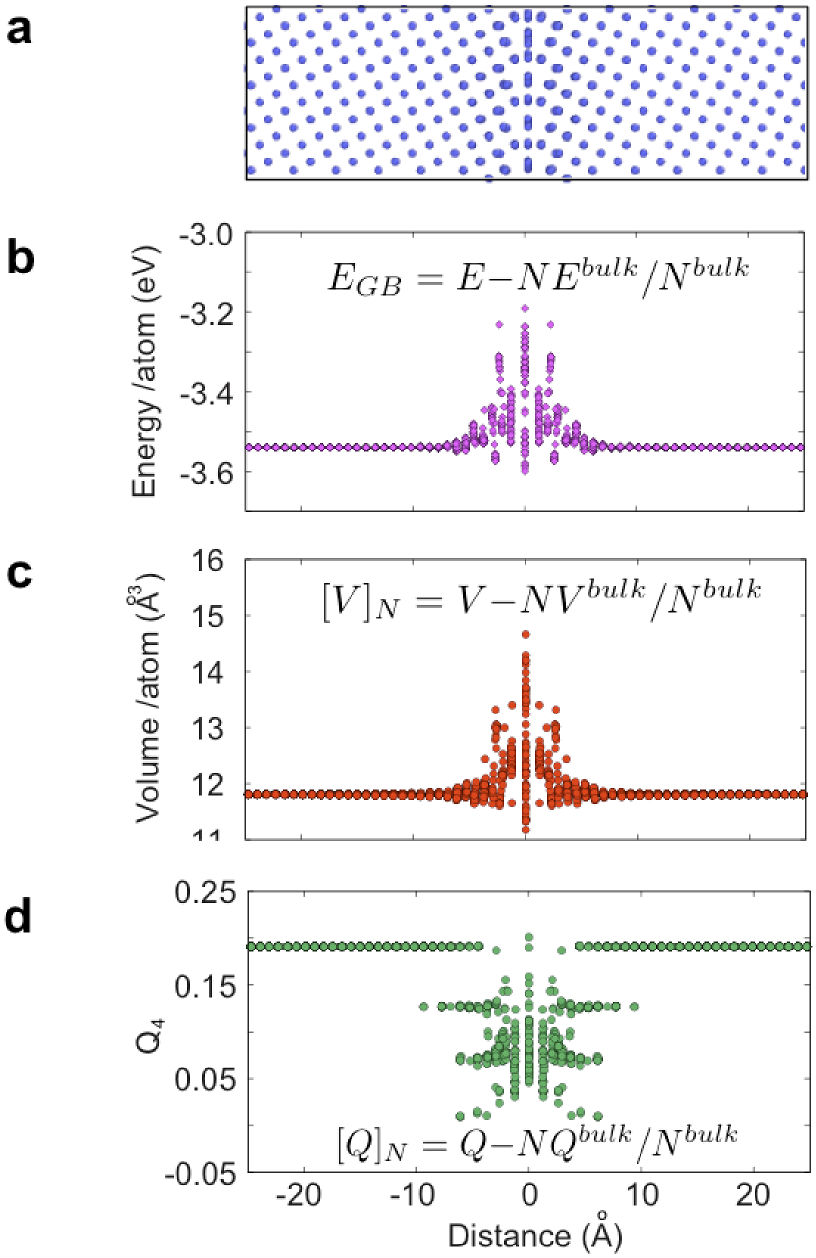

Supplementary Figure 6.

(a) A bicrystal with a grain boundary. (b) Energy per atom as a function distance normal to the boundary. (c) Volume per atom as a function distance normal to the boundary identified by Voronoi construction. (d) Q4 order parameter calculated for each atom as a function distance normal to the boundary. Properties in the boundary region are different from the bulk. These data can be used to calculate excess properties including $[E]_N$, $[V]_N$ and $[Q4]_N$ for each grain boundary structure.

Supplementary Note 1

Evolutionary algorithms (EAs) adopt concepts from evolutionary biology based on populations, selection, reproduction by heredity and mutation, aimed to locate the individual with highest fitness. The code generates a population of grain boundary structures and improves them over several generations to predict low-energy configurations. During the evolution complex and diverse structures with different atomic densities are sampled by operations of heredity and mutation which involve atomic rearrangements as well as insertion and removal of atoms from the grain boundary core. Although the model will be demonstrated on symmetric grain boundaries, the evolutionary algorithm also can be applied to asymmetric boundaries, as will be shown in future work. Supplementary Figure 4 shows the GB model and a schematic representation of the evolutionary algorithm. In our implementation, we split each GB model into three different regions, the region of upper grain (UG) and lower grain (LG), and grain boundary (GB). In the current implementation the UG-GB-LG model assumes periodic boundary conditions along GB plane and open boundaries perpendicular to the GB plane. If only 3-dimensional periodic boundary conditions are available in the ab-initio codes, a vacuum layer of 10-20 Å is added on top of UG, in order to eliminate the interaction between UG and LG.

Our optimization target is the atomic configuration in GB and the relative translation between UG and LG leading to the lowest GB energy. UG and LG regions are pre-specified and are typically 40-60 Å thick. The GB thickness is an input parameter pre-defined by the user. To ensure accurate GB energy calculation converged with the system size normal to the grain boundary plane, we sandwich the GB region by two buffer regions, approximately 20 Å for each direction. The atoms in the buffer zones are not affected by the evolutionary search, but can move freely during the energy minimization. GB energy is calculated as $\gamma_{\text{GB}} = E - E_{\text{coh}}N$, where E is the energy of the region with N atoms that includes both the GB slab and the buffer zones and E_{coh} is the energy per atom in the bulk Cu.

To initiate the evolutionary search, we create the first generation of structures with the grain boundary dimensions equal to random multiples of the periodic unit of the coincidence site lattice (CSL), which is dictated by the periodicity of the bulk crystals. The maximum GB dimension allowed in the search is a parameter specified by the user. A good strategy is to explore smaller GB cross-sections first. After the bicrystals are created, the GB slabs are populated with atoms by randomly by generating their positions while enforcing certain layer group symmetries. The layer group symmetries are also chosen at random for each individual GB structure.

Then we join UG-GB-LG together applying random translations between UG-GB, and GB-LG. The structures are then relaxed by performing an energy minimization using external computational codes, which can be based on empirical force fields such as in the current study or

ab-initio calculations. During this geometry optimization stage, the atoms in the GB slab and the buffer regions are relaxed, while the atoms in the LG are fixed and the atoms in the UG are treated as a rigid body. The constraint on the LG fixes the position of the bicrystal in space, while the relaxation and the rigid translation of the UG sets normal and shear stresses to zero. The energy minimization is followed by fitness evaluation, namely, the excess GB energy calculation. After that the parents are chosen from the top 60% of structures according to the tournament selection, which ensures a higher probability for GBs with higher fitness. The child structures for the next generation are produced in the following way: 1) *heredity,* which choses two GB structures and randomly slices them at the same position in the GB unit cell and then combines the pieces to generate the offspring; 2) *mutation* which choses one GB structure and displaces its GB atoms according to the stochastically picked soft vibrational modes based a bond-hardness model[1,2]; 3) *insertion/removal* of atoms, which changes the number of atoms in the GB slab. The offspring, together with 10% best structures from the previous generation, comprise the new population. This cycle is repeated until no lower-energy structures are produced for sufficiently many generations. The maximum number of generations is a parameter specified by the user and was set to 50 in this work. A typical run would explore the structures ranging from 500 to 5000 atoms for the entire model and 30 to 300 atoms for the GB region. A typical search for 50 generations takes about 1-3 days running on 1 core (3.00 GHz), with the majority of the computational cost spent on the geometry optimization and energy evaluation done by LAMMPS[3]. Below we expand on the key features of the evolutionary search.

GB structure initialization. Any pure random structure initialization or variation operation is very likely to lead to disordered, liquid-like structures with close energetics. Such parents are likely to produce similar children with poor fitness. To address this challenge, we followed the idea of coarse-grained modeling and define the simplified representations during the stage of structure generation. Symmetry has played a crucial role in the analysis of crystal structures and here we extended to grain boundaries. We propose a novel initialization scheme that generates the initial GB structures with the desired layer group (or space group)[2]. For each grain boundary a layer group symmetry is generated at random and is enforced during the initial population of the GB slab with atoms. Note that this symmetry could be broken or lowered by the subsequent variation operations like heredity and mutation. The number of atoms placed in each GB slab is estimated initially from the bulk density of the perfect crystal and the thickness defined by the user. This number is then randomly varied within the interval from 0 to N, where N is the number of atoms in one bulk atomic plane parallel to the GB. This ensures that structures with different atomic fractions are present in the initial population.

Mutation. We apply mutation in a reduced variable space: instead of displacing the atoms randomly or based on a Gaussian distribution, we calculate the vibrational modes corresponding to

both zero and nonzero wave vectors and displace the atoms along those soft modes (i.e., the vibrations with negative or small positive frequencies)[1]. The advantages are twofold. First, it mimics the structure transition due to phonon instability upon large elastic strain, thus is more likely to lead to child structure with low energy. Second, it naturally enables the cell size to spontaneously change during the simulation and thus could efficiently identify the optimum cell sizes due to structural modulation.

Large GB reconstructions. The exact dimensions of the grain boundary unit cell are not known prior to the search, which means that the calculations have to be performed for many different cross-section sizes and shapes. In prior studies[4] including our earlier work[5] large reconstructions such as $2{\times}5$ were found by systematic searches performed for a range of different cell sizes. The necessity to explore all possible GB sizes in addition to the optimization of the atomic structure makes the structure search prohibitively expensive. Instead of performing calculations for all possible cross-section sizes and shapes, we allow the GB dimensions to change automatically during the search. For heredity and insertion/removal of atoms operations, we first expand the parent structures to the new size by multiplying the simulation blocks. For mutations, we calculate the atomic displacements corresponding to both zero and nonzero wave vectors, enabling cell size to spontaneously change during the simulation[1].

Here, we illustrate the idea using the $\Sigma5(210)[001]$ GB as an example. In the first search, we set the maximum multiples of the CSL unit to be 6, which generates GBs with allowed dimensions of $1{\times}1$, $1{\times}2$, $1{\times}3$, $1{\times}4$, $1{\times}5$, $1{\times}6$, $2{\times}1$, $2{\times}2$, $2{\times}3$, $3{\times}1$, $3{\times}2$, $4{\times}1$, $5{\times}1$, $6{\times}1$ (the total of 14 different cross-sections). Initially, the different GB cross-sections are sampled with equal probability. However, after several generations some dimensions become more probable due to selection, since these dimensions are compatible with low-energy structures that have higher chance to survive. Supplementary Figure 5. illustrates the probability distribution of several different cross-sections of the $\Sigma5(210)[001]$ GB calculated over the entire search. The plot suggests that the dimensions such as $6{\times}1$, $5{\times}1$ and 4×1 are unlikely to lead to low energy structures. These dimensions were abandoned quickly during the evolution. On the other hand, other GB dimensions such as 3×2, $2{\times}2$, $1{\times}6$, $1{\times}5$ and others were encouraged to propagate. Several low-energy SK and FK structures were found in cells with dimensions $1{\times}3$, $1{\times}4$, $1{\times}5$, $1{\times}6$ and $2{\times}2$. This results of the search and the probability distribution in Supplementary Figure 5. suggest that the GB phases have large dimensions along the $[001]$ tilt axis. This information could be useful for further explorations of larger cross-sections. For instance, the subsequent runs with GB dimensions ($6 < m \times n <= 15$, $n = 1$) and ($6 < m \times n <= 15$, $n = 2$) , quickly identified the ground state structures of SK (1×15) and FK (2×7), as well as many other SK and FK like structures within about 2500 structure optimizations for each individual search.

Insertion/removal of atoms. Recently, the local degree of order was introduced to characterize

the quality of the environment and its symmetry for a given atomic position in the structure[2]. This concept turns out to be very useful to evaluate the contribution of each atom to the total energy. Therefore, it could serve as the basis for the fragment selection as well as insertion and removal of atoms in the EA variation operations. Atoms with the higher order should have a higher probability to be selected and lower probability to be deleted. To remove atoms from the GB slab, the algorithm first calculates the local order parameter for each atom in the region. The order parameter is described in Eq. (5) of Ref.[6]. A random fraction of atoms (not exceeding 25%) with the lowest degree of order is then deleted. To insert atoms into the GB slab, we identify sites unoccupied by atoms by constructing a uniform grid with a resolution of 1 $Å^3$ and fill them at random. To ensure relatively gradual changes in the GB structure, the random number of the inserted atoms also does not exceed 25% of the total number of atoms in the GB slab. It should be noted that both insertion/removal and heredity operations automatically involve the change in the number of atoms at the GB. To explore particular GB atomic densities, we have implemented constraint searches with the customized range of GB atomic densities specified as an input parameter provided by the user.

Supplementary Note 2

To compare different structures of the same grain boundary we calculated their excess properties. In this work each GB structure was characterized by eight excess properties. In a single component system grain boundary free energy γ is given by[7,8]

$$\gamma A = E - TS - \sigma_{33} V - \mu N = [E]_N - T[S]_N - \sigma_{33}[V]_N$$

where $[Z]_X$ are grain boundary excess properties expressed using Cahn's determinants[8,9]. Grain boundary free energy is a function temperature, stress and lateral strain as described by the adsorptions equation[7,10,11]

$$d(\gamma A) = -[S]_N dT - [V]_N d\sigma_{33} + \tau_{ij} A de_{ij}, \qquad i,j = 1,2$$

where e_{ij} is the elastic strain tensor. At 0 K we calculate excess volume $[V]_N$ and two components of grain boundary stress τ_{11} and τ_{11} as[9–11]

$$[V]_N = \frac{1}{A}(V - V^{\text{bulk}} N/N^{\text{bulk}})$$

$$\tau_{11} = [\sigma_{11} V]_N = \frac{1}{A}(\sigma_{11} V - \sigma_{11}^{\text{bulk}} V^{\text{bulk}} N/N^{\text{bulk}})$$

$$\tau_{22} = [\sigma_{22}V]_N = \frac{1}{A}(\sigma_{22}V - \sigma_{22}^{\text{bulk}}V^{\text{bulk}}N/N^{\text{bulk}})$$

The extensive properties V, $\sigma_{11}V$, $\sigma_{11}V$ and N are calculated inside an arbitrary chosen region containing grain boundary and portions of the bulk grains, while V^{bulk}, $\sigma_{11}^{\text{bulk}}V^{\text{bulk}}$, $\sigma_{11}^{\text{bulk}}V^{\text{bulk}}$ and N^{bulk} are calculated inside a region of a perfect bulk crystal. Notice that $V^{\text{bulk}}/N^{\text{bulk}} = \Omega$ is a volume per atom in the bulk. In atomistic simulations volume occupied by each atom was calculated by LAMMPS using the Voronoi construction[3]. The product $\sigma_{ij}V$ for each atom was also calculated by LAMMPS. In our calculations bulk stresses $\sigma_{11}^{\text{bulk}}$ and $\sigma_{22}^{\text{bulk}}$ are zero within the numerical accuracy.

In addition to the three thermodynamic quantities $[V]_N$, τ_{11} and τ_{22}, another feature that we used to compare different grain boundary structures is the quantity $[n]$ which we refer to as grain boundary atomic density[5]. This quantity is fundamentally different from excess volume $[V]_N$ and the two should not be confused. First, we calculate the total number of atoms in the system N and the number of atoms $N_{\text{plane}}^{\text{bulk}}$ in one atomic plane parallel to the GB and located inside the bulk part in the same system. $[n]$ is then calculated as the ratio $(N$ modulo $N_{\text{plane}}^{\text{bulk}})/N_{\text{plane}}^{\text{bulk}}$. Since it is measured as a fraction of $N_{\text{plane}}^{\text{bulk}}$, its value goes from 0 to 1. $[n]$ is also a periodic quantity: an insertion of a perfect atomic plane with $N_{\text{plane}}^{\text{bulk}}$ atoms results in the same grain boundary structure. Due to the periodicity, the atomic density distance between two structures a and b was calculated as $min(abs([n^a] - [n^b]), 1 - abs([n^a] - [n^b]))$.

In addition to the four features described above we introduced grain boundary excess amounts of Steinhardt order parameters Q4, Q6, Q8 and Q12[12]. These parameters per atom are calculated within LAMMPS[3]. The grain boundary excess amounts of these parameters per unit area are then introduced in a manner analogous to the thermodynamic excess properties.

$$[Q]_N = \frac{1}{A}(Q - Q^{\text{bulk}}N/N^{\text{bulk}})$$

where $Q = \sum_{i=1}^{N} Q^i$ is the total amount of the order parameter in a region enclosing the grain boundary and containing N atoms, $Q^{\text{bulk}}/N^{\text{bulk}}$ is the value of this order parameter per atom in the bulk. Q is one of the Q4, Q6, Q8 or Q12. Per atom properties involved in the GB excesses calculations are illustrated in Supplementary Figure 6.

Supplementary References

[1] Zhu, Q., Oganov, A. R., Lyakhov, A. O. & Yu, X. Generalized evolutionary metadynamics for sampling the energy landscapes and its applications. *Phys. Rev. B* **92**, 024106 (2015).

[2] Lyakhov, A. O., Oganov, A. R., Stokes, H. T. & Zhu, Q. New developments in evolutionary structure prediction algorithm USPEX. *Comput. Phys. Commun.* **184**, 1172 – 1182 (2013).

[3] Plimpton, S. Fast parallel algorithms for short-range molecular-dynamics. *J. Comput. Phys.* **117**, 1–19 (1995).

[4] Chua, A. L. S., Benedek, N. A., Chen, L., Finnis, M. W. & Sutton, A. P. A genetic algorithm for predicting the structures of interfaces in multicomponent systems. *Nat Mater* **9**, 418–422 (2010).

[5] Frolov, T., Olmsted, D. L., Asta, M. & Mishin, Y. Structural phase transformations in metallic grain boundaries. *Nat. Commun.* **4**, 1899 (2013).

[6] Lyakhov, A. O., Oganov, A. R. & Valle, M. How to predict very large and complex crystal structures. *Comput. Phys. Commun.* **181**, 1623 – 1632 (2010).

[7] Gibbs, J. W. *The Scientific Papers of J. Willard Gibbs*, vol. 1 (Longmans-Green, London, 1906).

[8] Cahn, J. W. Thermodynamics of solid and fluid surfaces. In Johnson, W. C. & Blackely, J. M. (eds.) *Interface Segregation*, chap. 1, 3 (American Society of Metals, Metals Park, OH, 1979).

[9] Frolov, T. & Mishin, Y. Temperature dependence of the surface free energy and surface stress: An atomistic calculation for cu(110). *Phys. Rev. B* **79**, 045430 (2009).

[10] Frolov, T. & Mishin, Y. Thermodynamics of coherent interfaces under mechanical stresses. i. theory. *Phys. Rev. B* **85**, 224106 (2012).

[11] Frolov, T. & Mishin, Y. Thermodynamics of coherent interfaces under mechanical stresses. ii. application to atomistic simulation of grain boundaries. *Phys. Rev. B* **85**, 224107 (2012).

[12] Steinhardt, P. J., Nelson, D. R. & Ronchetti, M. Bond-orientational order in liquids and glasses. *Phys. Rev. B* **28**, 784–805 (1983).
